# NOS1-, NOS3-, PIK3CA-, and MAPK-pathways in skin following radiation therapy

**DOI:** 10.1186/s40364-017-0084-9

**Published:** 2017-01-20

**Authors:** Steffen Koerdt, Nadine Tanner, Niklas Rommel, Nils H. Rohleder, Gesche Frohwitter, Oliver Ristow, Klaus-Dietrich Wolff, Marco R. Kesting

**Affiliations:** 1 Department of Oral and Maxillofacial Surgery, Technical University of Munich (TUM), Ismaninger Str. 22, D-81675 Munich, Germany; 2 Department of Oral and Maxillofacial Surgery, Heidelberg University Hospital, Im Neuenheimer Feld 400, D-69120 Heidelberg, Germany

**Keywords:** Radiation therapy, MAPK, NOS, PIK3CA, PCR

## Abstract

**Background:**

Essential molecular pathways such as the MAPK pathway, NO system, or the influence of PIK3CA as an oncogene are known to regulate fundamental signalling networks. However, few knowledge about their role in the occurrence of wound healing disorders (WHD) following radiation therapy (RT) exists. This study aims to evaluate the expression profiles of specific molecular pathway marker genes.

**Methods:**

Expression profiles of the genes encoding MAPK, NOS1, NOS3, and PIK3CA were analyzed, by RT-PCR, in specimens from patients with and without a history of RT to the head and neck. Clinical data on the occurrence of cervical WHDs were analyzed.

**Results:**

Expression analysis of patients with postoperative WHDs revealed a significant increase in MAPK expression compared to the control group without occurrence of postoperative WHDs. PIK3CA showed a significantly increased expression in patients with a history of RT. Expression analysis of all other investigated genes did not reveal significant differences.

**Conclusions:**

This current study is able to show the influence of RT on different molecular pathways. This underlines the crucial role of specific molecular networks, responsible for the occurrence of long-term radiation toxicity such as WHDs. Additional studies should be carried out to identify possible starting points for therapeutic interventions.

## Background

In an interdisciplinary and individual oncological treatment concept for cancers of the oral cavity, Radiation Therapy (RT) is next to the surgical resection, and chemotherapy one of the most important columns for local and regional disease control. However, RT is also accompanied with multiple side effects, which can be subdivided in short- and long-term radiation associated toxicity. In cases, when patients received RT preoperatively in a neo-adjuvant setting or in cases of disease recurrence, surgeons have to deal with wound healing disorders (WHD) in previously irradiated tissue [1]. Factors, which might influence the onset of WHDs after RT appear to be immanent in terms of a reduced angiogenesis and the development of radiation-induced skin fibrosis [2–4]. Reactive oxygen and nitrogen species (RONS) in the context of oxidative stress also seem to play an important role in the pathogenesis of post-radiogenic WHDs, yet more research has to be conducted to fully understand the underlying molecular pathways and to find starting points for therapeutic interventions [5]. This current PCR-based study aims to evaluate the role of certain crucial pathways in the development of post-radiogenic WHDs following RT to the head and neck.

Nitric oxide (NO) is considered to be a short-living radical, which is involved in many important biological functions, such as vasodilation, anti-microbial activities, immunoregulation, and activities in which it functions as a neurotransmitter [6–10]. Its relevance in wound healing has been described elsewhere [11, 12]. NO is produced by nitric oxide synthase (NOS) in different isoforms. Neuronal NOS (NOS1) and endothelial NOS (NOS3), collectively referred to as cNOS are constitutively expressed depending on intracellular calcium levels [11]. The influence of comorbidities such as protein calorie malnutrition, diabetes, and steroid use, which might be accompanied with impaired wound healing have all been shown to be associated with an reduced NOS expression [13, 14]. Wang and co-workers were able to show, that NO production by dermal fibroblasts could be important during inflammatory stages of wound healing after skin injury [15].

The catalytic p110 subunit of class one phosphatidylinositol 3-kinase (PIK3CA) regulates pathways important for cell proliferation, survival, and motility [16, 17]. Samuels et al. were able to identify PIK3CA as an oncogene, with capacities as a useful marker for detection of cancers and for monitoring tumor progression [18]. The effects of UV exposure on PIK3CA mutations in skin have been described in the context of the pathogenesis of solar lentigo and benign lichenoid keratosis [19, 20].

Moreover, reports from the current literature suggest, that UVA radiation can activate mitogen-activated protein kinase (MAPK) pathway [21]. Its activation leads to activator protein-1(AP-1) induction and consequently the regulation of matrix metalloproteinase (MMP) genes. The breakdown of dermal collagen in photo-related aging has been shown to be associated with UV-induced MMPs expression, also being regulated by underlying MAPK-pathways [22, 23].

However, no distinct reports about of the influence therapeutic ionizing radiation on the MAPK pathway, NO signalling system, or PIK3CA as an essential oncogene exist in the current literature. All pathways and underlying regulatory functions build a complex network responsible for fundamental cell characteristics such as proliferation and apoptosis. This study aims to evaluate the expression of NOS isoforms, PIK3CA, and MAPK in a PCR-based study. Further understanding of essential pathways and corresponding turning points might add up to the knowledge about radiation-induced toxicity.

## Methods

This study followed the Declaration of Helsinki on medical protocol and ethics, and the Institutional Review Board of the Technical University of Munich (TUM), Germany approved the study (No. 307/11). All participants were informed extensively and signed an informed consent agreement.

All patients, who met the following inclusion criteria were recruited for the study. Potential patients had to be in-patients at the Department of Oral and Maxillofacial Surgery, Technical University of Munich (TUM), Germany between January 1^st^, 2012 and December 31^st^, 2012. All study patients had to be older than 18 years, anamnestic data had to be available, and tissue specimens from the surgical access to the neck had to be available for further experiments. Clinical data (age, sex, history of RT, history of tobacco/alcohol abuse, etc.) were obtained and documented before surgery. During hospitalization, clinical data on the occurrence of cervical WHD was obtained at an interval of 30 days postoperatively.

Tissue samples from the neck were obtained from incision margins during the surgical access with approximate dimensions of 1 × 5 mm. Specimens were stored in Allproctect^™^-Solution (Qiagen, Hilden, Germany) at −80 °C for PCR analysis.

### RT-PCR

Ribonucleic acid (RNA) isolation was performed by using the RNeasy® Protect Mini Kit (Qiagen). Beforehand, tissue samples were comminuted by using a rotor-stator system (Miccra, ART Labortechnik, Müllheim, Germany) and ultrasonification. After measurement of the amount of extracted RNA by means of a Biophotometer (Eppendorf, Hamburg, Germany), 1 μg isolated RNA was used for reverse transcription. Reverse transcription was performed according to the protocol of the SuperScript™ First Strand Synthesis System (Invitrogen, Carlsbad, USA). Random primers were used for RT.

For RT-PCR, 2 μl cDNA sample, 2 μl LightCycler® FastStart DNA Mater SYBR Green I reaction mix (Roche, Mannheim, Germany), 1 μl forward and reverse primers (0.5 μmol L^−1^), 1.6 μl MgCl_2_ (3 mmol L^−1^), and 12.4 μl RNase-free water, resulting in a volume of 20 μl/sample were analyzed by using the LightCycler® 1.0 system (Roche). Primer specificity was tested by using electrophoretic separation of the PCR product. Primer specifications are shown in Table 1. Amplification algorithms were as follows: 10 min at 95 °C, 40 cycles of 15 s at 94 °C, 10 s at 60 °C, and 10 s at 72 °C. A melting curve analysis was recorded in order to test for cDNA fragment consistency. The amount of RNA was automatically calculated by comparison of measured threshold cycles with standard curves and normalized with glyceraldehyde 3-phosphate dehydrogenase (GAPDH) as a housekeeping gene. A no template control was included in each run. All amplifications were carried out in triplicates.

**Table 1:** Primer sequences of examined genes

Gene	Sequence 5’to 3	
MAPK	Forward	CAGTGGGATGGAATTGAAAG
	Reverse	AGCAGAAGGAATGAGTGTGC
NOS1	Forward	GACTGTTGAGATGGAAGAAC
	Reverse	ATCTTCCTGTCTCCGAGGCG
NOS3	Forward	GTGATGGCGAAGCGAGTGAAG
	Reverse	CCGAGCCCGAACACACAGAAC
PIK3CA	Forward	CTCCACGACCATCATCAGG
	Reverse	GATTACGAAGGTATTGGTT

### Statistical analysis

All data were analyzed by using IBM®SPSS® for Mac (version 22.0; IBM Corp., USA). Means and standard deviation (SD) were calculated, and tests of significance were performed. For normally distributed values, *t*-test was performed. For values not normally distributed, the Mann–Whitney test was used. Statistical significance was defined as *α* = 0.05. All *p*-values are local and given as two-tailed.

## Results

A total of 30 patients were enrolled in the current study. Nineteen (63.3%) were male, 11 (36.7%) of were females. The mean age was 57.1 ± 10 years (minimum 43, maximum 76 years). Fifteen patients (50%) received a radiation to the head and neck at least six months before the surgical intervention took place and the tissue specimens were obtained. Patients with a history of RT were exposed to dosages of a mean of 60.0 ± 6.32 Gy (Gy) (Maximum 69.9 Gy; Minimum 50.0 Gy). Alcohol abuse could be observed in 13 patients (43.3%), nicotine abuse was observed in 19 patients (63.3%).

**Fig. 1:**
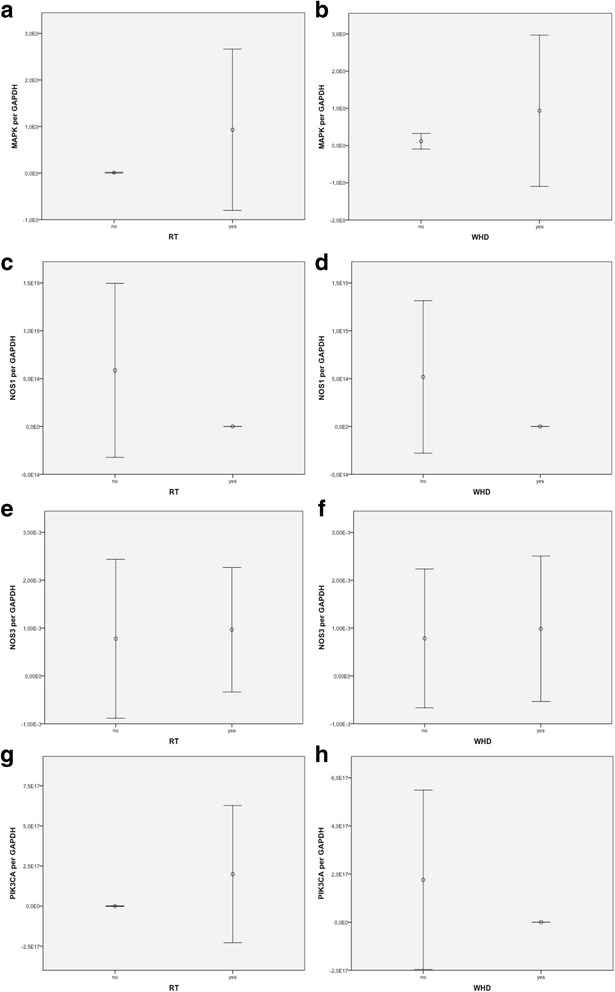
Bar graph visualizing visualising the expression of the investigated genes MAPK in patients with and without a history of Radiation Therapy (RT) **a**, as well as in patients with and without the occurrence of postoperative Wound Healing Disorders (WHD) **b**. Expression levels of NOS1 **c**, **d**, NOS3 **e**, **f**, and PIK3CA **g**, **h** are displayed accordingly. Measured by real-time RT-PCR in cervical tissue samples. Given is the median value, with error bars indicating the 95% confidence interval (CI)

The results of RT-PCR analysis of the expression of MAPK are displayed in Fig. 1a and b and Table 2. No statistical significant difference could be observed in context of a preoperatively irradiated tissue (*p* = .263). Expression analysis of patients with postoperative WHDs revealed a significant increase in MAPK expression compared to the control group without occurrence of postoperative WHDs (*p* = .043). Expression of NOS1 was decreased in irradiated tissue without statistical significance (*p* = .178; Fig. 1c, Table 2). In analysis of tissue samples from patients with postoperative WHDs, a decrease of NOS1 expression could be observed (*p* = .241; Fig. 1d, Table 2). Analysis of expression profiles of NOS3 revealed increase in previously irradiated tissue (*p* = .089; Fig. 1e, Table 2) and in tissue samples from patients with postoperative WHDs (*p* = .680; Fig. 1f, Table 2). PIK3CA showed a significantly increased expression in tissue samples from patients with a history of RT (*p* = .049; Fig. 1g, Table 2). In analysis of tissue with occurrence of postoperative WHDs, an increase in samples without WHDs became obvious, even though no statistical significance could be detected (*p* = .391; Fig. 1h, Table 2).

**Table 2:** Real-time RT-PCR results in groups with/without preoperative RT and with/without postoperative cervical WHDs

Gen	Median Expression per GAPDH			Median Expression per GAPDH		
	RT	No RT		WHD	No WHD	
	N (%)	N (%)	*p* Value	N (%)	N (%)	*p* Value
MAPK	9.32 E-01	9.65 E-03	.263	9.36 E-01	1.15 E-01	.043*
	15 (50)	15 (50)		13 (43.3)	17 (56.7)	
NOS1	6.65 E-05	5.86 E + 14	.178	7.67 E-05	5.173 E + 14	.241
	15 (50)	15 (50)		13 (43.3)	17 (56.7)	
NOS3	9.66 E-04	7.77 E-04	.089	9.86 E-04	7.84 E-04	.680
	15 (50)	15 (50)		13 (43.3)	17 (56.7)	
PIK3CA	1.99 E + 17	2.16 E + 06	.049*	6.48 E + 03	1.76 E + 17	.391
	15 (50)	15 (50)		13 (43.3)	17 (56.7)	

Abbreviations: *WHD* wound healing disorder; *RT* radiation therapy; *RT-PCR* reverse-transcription polymerase chain reaction

Data in parentheses are percentages, unless noted otherwise; *statistically significant difference at *α* = 0.05

## Discussion

In an interdisciplinary and individual treatment concept in head and neck malignancies, next to the operative resection, RT with or without a concomitant chemotherapy is one of the major columns. In a clinical setting, patients with a history of RT, who are in need of a surgical intervention, regularly present with WHDs in the application field of RT. These effects have been studied before and the influence of RT on wound healing has been described elsewhere [1–5]. Impaired wound healing is regarded to be one of the long-term side effects of RT, amongst others. However, this poses to be an increasing surgical challenge, as an increasing amount of patients with previous conservative treatments present with the need for a surgical intervention, because of tumor recurrence, local metastases, or the growth of a second primary tumor. Fundamental signalling pathways seem to play an important role in the clinical occurrence of long-term side effects related to RT. These pathways and their regulatory influence on the occurrence of post-radiogenic WHDs in particular still remain subject to further research. The identification of sign posts in this complicated network of signalling pathways could not only help to further understand the underlying mechanisms of radiation induced side effects, but might also offer starting points for therapeutic interventions in the future in order to reduce RT related side effects or to facilitate the treatment of specific side effects such as WHDs. This current study aims to evaluate different signalling pathways in previously irradiated human tissue specimens and those without any influence of ionizing radiation in a PCR-based setting.

MAPK was increased in irradiated tissues and in tissue specimens, which developed WHDs postoperatively. Kim et al. as well as Shin and colleagues described the influence of MAPK-pathways in photo-related skin aging by UV-induced MMPs expression [22, 23]. Moreover, the influence of ionizing radiation on the MAPK pathway seems to be definite, even though this study could not show any significant differences. The role of MAPK in the occurrence of radiogenic long-term toxicity should definitely be subject to further research. However, its impact could be described in this current investigation.

Evaluation of NOS systems in irradiated skin revealed an increase of NOS1 in not previously irradiated tissue and tissue specimens without occurrence of postoperative WHDs, whereas NOS3 was expressed contrariwise, however no significant differences could be observed in statistical analysis for both isoforms. NOS1 and NOS3 are together being referred to as cNOS. Influence of cNOS expression through various comorbidities has been described in the literature [13, 14]. Nevertheless, this current study describes the influence of therapeutic dosages of ionizing radiation on expression levels of cNOS. Furthermore, we were able to show that NOS1 and NOS3 seem to act contradictory.

Analysis of expression levels of PIK3CA as an essential oncogene showed a significant increase in irradiated tissues, whereas no significance could be detected in sub-analysis of specimens with or without the occurrence of postoperative WHDs. The ability of PIK3CA of detecting neoplasms, its usefulness in monitoring tumor progression, as well as the influence of UV exposure on PIK3CA mutations have been subject to extensive research [18–20]. This investigation describes the influence of RT on expression levels of PIK3CA. However, gene amplifications of PIK3CA have been studied in head and neck squamous cell carcinomas by Qiu and colleagues [24]. The oncogenic properties of PIK3CA in the carcinogenesis of head and neck cancers could be described. This might be of special interest as all specimens in this current study were derived from patients with head and neck cancers. Nevertheless, as no significantly increased expression levels were found this fact seems to be crucial for carcinogenesis but not for RT associated side effects.

Taking the results of the current study into consideration, we were able to show the influence of RT on different molecular pathways. However, the influence of specific pathways in therapeutic RT and especially in long-term radiation-related side effects still remains subject to further research, this data underlines the crucial role of molecular pathways. Nevertheless, additional studies need to be carried out to identify possible starting points for novel therapies.

## Conclusions

This current study is able to show the influence of RT on different molecular pathways. This underlines the crucial role of specific molecular networks, responsible for the occurrence of long-term radiation toxicity such as WHDs. Additional studies should be carried out to identify possible starting points for therapeutic interventions.
